# Effects of Consuming a Low Dose of Alcohol with Mixers Containing Carbohydrate or Artificial Sweetener on Simulated Driving Performance

**DOI:** 10.3390/nu10040419

**Published:** 2018-03-28

**Authors:** Bryce Brickley, Ben Desbrow, Danielle McCartney, Christopher Irwin

**Affiliations:** Menzies Health Institute Queensland, School of Allied Health Sciences, Griffith University, Gold Coast 4215, Australia; b.desbrow@griffith.edu.au (B.D.); danielle.mccartney@griffithuni.edu.au (D.M.); c.irwin@griffith.edu.au (C.I.)

**Keywords:** simulated driving, driving, alcohol, carbohydrate, artificial sweetener, cognitive performance

## Abstract

The Australian National Drug and Alcohol Research Centre (NDARC) devised gender-based drinking recommendations to ensure blood or equivalized breath alcohol concentrations (BrAC) remain <0.050%. However, these may be inappropriate for individuals consuming alcohol without carbohydrate (CHO), which results in higher BrACs. This study investigated the effects of ingesting alcohol with and without CHO on BrACs and simulated driving performance. Thirty-two participants (16 males; age: 23 ± 6 years) completed two randomized single-blinded trials. Participants performed a baseline drive (Drive 1), then an experimental drive (Drive 2), following alcohol consumption (males: 20 g; females: 10 g). Alcoholic beverages contained either 25 g sucrose or aspartame (AS). Driving performance was assessed using lateral control (standard deviation of lane position [SDLP] and number of lane departures) and risk-taking (number of overtaking maneuvers and maximum overtaking speed). BrAC and subjective ratings (e.g., intoxication) were also assessed. BrAC was significantly lower as Drive 2 commenced with CHO compared to AS (0.022 ± 0.008% vs. 0.030 ± 0.011%). Two males provided BrACs >0.050% with AS. Neither beverage influenced changes to simulated driving performance. Ingesting alcohol in quantities advised by the NDARC results in no detectable simulated driving impairment. However, the likelihood of exceeding the legal drink-driving BrAC is increased when alcohol is consumed with artificially-sweetened mixers.

## 1. Introduction

Alcohol is implicated in ~34% of fatal motor vehicle crashes each year in Australia [1], with similar rates observed among other developed nations (e.g., New Zealand (29%) [2], the US (31%) [3] and the UK (14%) [4]). Thus, alcohol-involved road traffic crashes represent a significant source of unintentional injury and mortality [5]. Impairment of driving-related skills (e.g., delayed reaction time and reduced vigilance) commences at the lowest measurable blood and equivalized breath alcohol concentrations (BrACs) (i.e., >0.000% BrAC) and increases in a dose-dependent manner thereafter [6]. As a result, statistically, crash risk is significantly elevated above 0.040% BrAC and increases exponentially above 0.100% BrAC [7].

To reduce the incidence of road traffic crashes, many countries enact legislation that prohibits or restricts driving under the influence of alcohol [8]. In Australia, state and territory governments enforce a maximum legal driving limit of 0.050% BrAC for open class (fully-licensed), non-professional drivers [9]. Thus, alcohol consumers are required to estimate BrAC based on drinks being consumed. However, consumers have typically demonstrated a limited capacity to accurately estimate BrAC [10,11]. The National Drug and Alcohol Research Centre (NDARC) has devised gender-based recommendations to ensure individuals remain below the 0.050% BrAC threshold when choosing to consume alcohol [12]. These recommendations stipulate that females are able to consume ≤1 standard drink (10 g alcohol) in the first hour of drinking, whilst males are able to consume ≤2 standard drinks (20 g alcohol). However, a number of factors (e.g., drinking history, body composition and beverage type) can influence alcohol pharmacokinetics [13]. Hence, the ability of these recommendations to maintain BrAC <0.050% is likely to be highly contextual.

Consuming alcohol with mixers (non-alcoholic beverages) that contain carbohydrate has been demonstrated to attenuate BrAC [14,15,16,17,18]. Indeed, a recent investigation in females observed a 37% reduction in peak BrAC when alcohol was ingested with 50 g of carbohydrate (CHO), compared to artificial sweeteners (AS) (0.038 ± 0.011% vs. 0.052 ± 0.007% BrAC); a smaller reduction (8%) was noted with a lower dose of CHO (15 g) [14]. In addition, it appears that individuals are unable to detect differences in subjective feelings of intoxication when the CHO content of an alcoholic beverage is manipulated [14,16]. Therefore, ingesting alcohol in the absence of CHO may increase the likelihood of exceeding the 0.050% BrAC limit and one’s perception of safety to operate a motor vehicle. These findings are particularly relevant as the consumption of alcoholic beverages containing AS appears to be gaining popularity [19], with the behavior thought to be motivated by a desire to reduce caloric intake and/or enhance the intoxication effects of alcohol [20].

To date, two studies have investigated the influence of co-ingesting alcohol with CHO or AS on cognitive performance [14,16]. These studies failed to observe differences in choice reaction time (CHO BrAC = 0.038 ± 0.011 vs. AS BrAC = 0.052 ± 0.007%) [14] and reaction time on a cued go/no-go task (CHO BrAC = 0.070 ± 0.012 vs. AS BrAC 0.079 ± 0.015%) [16] between treatments. However, the earlier investigation [16] did note significant impairment of cued go/no-go reaction time when alcohol was consumed with AS compared to a placebo alcohol beverage (no such impairment was observed when the CHO treatment was compared against the alcohol-placebo). Hence, it is conceivable that alcohol-induced impairment of cognitive function may be attenuated in the presence of CHO. Importantly, the aforementioned studies both targeted relatively simple cognitive abilities using discrete cognitive tasks. Complex tasks that require individuals to simultaneously co-ordinate various interdependent cognitive processes (e.g., when operating a motor vehicle) may be more sensitive to detecting differences between treatments (i.e., alcohol + CHO vs. alcohol + AS).

Therefore, the aim of the present study was to investigate the effects of co-ingesting alcohol (in accordance with the NDARC dosing recommendations for males and females) with and without CHO on simulated driving performance. It was hypothesized that the presence of CHO in an alcoholic beverage would result in lower BrAC responses and attenuate alcohol-induced impairment in simulated driving performance. The results of this investigation could have important implications for public health recommendations provided to individuals who plan to drive following alcohol ingestion.

## 2. Materials and Methods

### 2.1. Participants

To be considered eligible to participate in this study volunteers were required to be aged ≥18 years and to have held a provisional/open Australian driving license for ≥2 years. The number of participants was determined following a sample size calculation for one common simulated driving outcome measure—standard deviation of lane position (SDLP)—using power calculation software (G*Power Version 3.1.9.2, University Kiel, Germany, 2014). Kenntner-Mabiala et al. [21] detected a 3.0 ± 1.1 cm increase in SDLP under a low-dose alcohol treatment (0.050% BrAC) vs. placebo with an effect size of 0.6 [22]. Using this effect size, a power (1 − β) of 0.9 and α = 0.05 (for *a priori* planned comparisons), the estimated total sample required was *n* = 32. Thirty-three individuals volunteered to take part in this study. One participant withdrew from the study after completing the first experimental trial due a musculoskeletal injury (unrelated to this study). The remaining 32 participants (16 males; age: 22.9 ± 5.8 years; weight: 71.2 ± 11.3 kg; body mass index (BMI): 23.8 ± 3.0 kg·m^−2^) had consumed alcohol regularly for 4.7 ± 5.5 years and typically spent 1.1 ± 0.7 h·day^−1^ driving. Participants were categorized as low (*n* = 14), medium (*n* = 15) and high (*n* = 3) risk takers, in accordance with the RT-18 screening tool [23]. The mean risk taking factor was 7.4 ± 3.9 (medium risk-taking tendency) [23]. This investigation was approved by the University’s Human Research Ethics Committee (GU Ref No.: 2017/046) and the procedures were conducted in accordance with the principles outlined by the agreement of Helsinki.

### 2.2. Study Design

**Figure 1:**
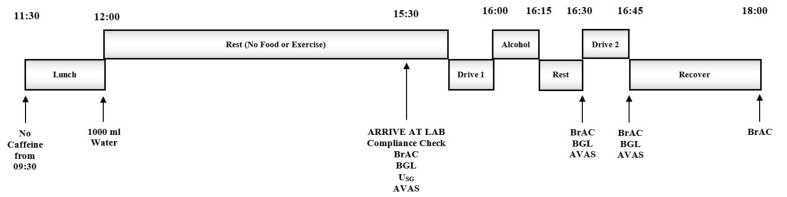
Experimental Protocol. U_SG_ = Urine Specific Gravity, BrAC = Breath Alcohol Concentration, BGL = Blood Glucose Level, AVAS = Subjective Ratings (Adaptive Visual Analog Scales).

This single-blind randomized controlled study utilized a repeated-measures design. Participants completed two experimental trials counterbalanced for order (using an incomplete Latin square) and separated by >24 h. During each trial, individuals undertook a baseline simulated drive (Drive 1) before completing an experimental simulated drive (Drive 2) following alcohol consumption. The alcohol (males: 20 g; females: 10 g, as per NDARC recommendations [12]) was administered as vodka (37.5% *v*/*v*) mixed with soda water and either 25 g sucrose (CHO) or artificial sweetener (AS), matched for sweetness. Breath alcohol concentration (BrAC), blood glucose level (BGL) and subjective ratings (e.g., intoxication, impairment) were measured periodically throughout the experimental protocol (Figure 1). 

#### 2.2.1. Preliminary Screening

Volunteers presented to the laboratory where eligibility to participate was assessed. First, individuals completed a health assessment questionnaire to determine medical history and drinking habits. Those who were pregnant, diabetic, and/or self-reported a psychiatric disorder, head trauma or other central nervous system injury were excluded from the study. Individuals were also excluded if they indicated recent use (i.e., <6 months prior) of recreational drugs and/or psychoactive medications (e.g., benzodiazepines). The Short Michigan Alcoholism Screening Test (SMAST) was used to identify disordered alcohol use; individuals scoring ≥5 on the SMAST were ineligible to participate [24]. Risk-taking tendencies were evaluated via the RT-18 screening tool [23]. BMI was imputed from height and weight measurements taken using digital scales (SECA 804 digital scales, ECOMED Trading Pty Ltd., Seven Hills, NSW, Australia) and a stadiometer. Volunteers with a BMI >30 kg·m^−2^ or <18.5 kg·m^−2^ were excluded to minimize variation in body composition across the participant sample. Eligible participants performed a 5 min familiarization with the driving simulator to reduce practice effects [25].

#### 2.2.2. Pre-Experimental Procedures

Participants were instructed to limit their alcohol intake to ≤2 standard drinks on the evening prior to each trial. On trial days, individuals were required to avoid caffeinated foods and beverages after 9:30 a.m., to consume lunch and 1000 mL of water between 11:30 a.m. and 12:00 p.m. and to fast from all food and fluid (except water) until they arrived at the laboratory ~3.5 h later (15:30 p.m.). A record of food consumed at lunch was obtained at the first trial; participants were asked to replicate this diet before the subsequent trial. Compliance to these procedures was verbally confirmed at the beginning of each trial.

#### 2.2.3. Experimental Procedures

On arrival at the laboratory, participants provided a breath sample using a police grade portable breathalyzer (Alcolizer LE4; Alcolizer Technology, Cleveland, QLD, Australia) to verify a zero BrAC and provided a urine sample to determine urine specific gravity (U_SG_) as a marker of hydration status (Refractometer UG-α^®^; Atago Co., Ltd., Tokyo, Japan). Participants who produced a urine sample indicating some level of dehydration (U_SG_ > 1.024 [26]) were given ~700 mL of water to consume before U_SG_ was reassessed 30 min later. The procedure was repeated until a urine sample below the accepted threshold for euhydration (U_SG_ of <1.024) was obtained. Participants then provided a finger-prick blood sample to confirm they were not hypoglycemic (BGL ≤ 3.5 mmol·L^−1^) (Accuchek Advantage II; Roche, Castle Hill, NSW, Australia). The Stanford Sleepiness Scale [27] and a series of visual analog scales were used to indicate alertness and mood/arousal. Participants then performed a 15 min simulated driving task (Drive 1) before consuming alcohol and repeating the simulated driving task (Drive 2). BrACs and BGLs were assessed at the cessation of drinking and at the onset and conclusion of Drive 2. Participants rated their mood/arousal and impairment/intoxication at the onset of Drive 2 and reported their perceived driving performance at the end of Drive 2. All BrAC and BGL measurements were taken in duplicate and concealed from participants. On conclusion of the final experimental trial, participants completed a questionnaire to determine the effectiveness of the blinding procedures, assess individual expectations in regard to the influence of consuming alcohol with/without CHO and evaluate perceptions of the simulated driving task.

### 2.3. Treatment Beverages

The alcoholic beverages were administered as vodka (37.5% *v*/*v* VodkaO^™^; Artisan Spirit Merchants, Melbourne, VIC, Australia) mixed with soda water (~4 °C) and either 25 g sucrose (CHO) (CSR Sugar, Yarraville, VIC, Australia) or artificial sweetener (AS) (Ajinomoto pure aspartame; Melbourne Food Depot, Melbourne, VIC, Australia). The alcohol was dosed as per the NDARC recommendations [12], with males receiving two standard alcoholic beverages and females receiving one (i.e., 10 g alcohol⋅ standard drink^−1^). Participants were instructed to consume the beverage(s) at an even pace over 15 min and rest for a further 15 min before beginning the simulated driving task (Drive 2). Post-drive BrAC readings were taken regularly until BrAC was <0.010%, at which point subjects were permitted to leave the laboratory.

### 2.4. Driving Simulator Apparatus

Details of the driving simulator apparatus have been outlined elsewhere [25]. Briefly, the simulator included a fixed-based model with standard automatic controls (accelerator and brake pedals, steering wheel, seat, safety belt, indicator, automatic gear shift and hand brake) from a real vehicle. The in-built software of the simulator (SCANeR studio simulation engine, v1.6r50, OKTAL, Paris, France) automatically recorded performance data, which was converted to a data spreadsheet for subsequent analysis.

### 2.5. Simulated Driving Task

The itinerary-defined course (17 km, ~15 min duration) consisted of a single-lane, bidirectional road (4.0 m lane width) with some hills (incline: 0.5–6.6%) and bends (radii: 123–1870 m). The scenery reflected an urban road environment (i.e., including buildings and trees). Participants were instructed to adhere to Australian road rules and drive in the center of their lane. The roadway was divided into 3 main segments. Vehicle control was assessed in two distinct segments of the course, separated by a “risky-driving” section of the course. Light oncoming traffic programmed to exhibit non-conflicting behavior was present in these sections. Speed restrictions varied between 60 and 80 km·h^−1^ and were signed accordingly. Driving performance was assessed as lateral control (SDLP_1_, SDLP_2_, SDLP_Total_ (i.e., SDLP across Section 1 and Section 2) and the total number of side and center lane crossings [LC]). SDLP refers to the driver’s ability to maintain lane position, which is increased by lateral movement; thus, increased SDLP increases the likelihood of LC [28]. This measure often indicates lane swerving behaviors commonly exhibited by intoxicated drivers and is often used as a measure of driver safety in simulated driving research [28]. Both SDLP and LC recorded during simulated driving have demonstrated sensitivity to the intoxication effects of low doses of alcohol (BrAC ≤ 0.050%) [22]. SDLP was computed by calculating the standard deviation of the mean lateral position of the driver’s vehicle (relative to the center of the driving lane) across the entire driving scenario, as described by Verster and Roth [29]. Risky driving behavior was assessed in a separate segment of the course. Two cars and a truck were positioned at different locations on the roadway (ahead of the participants’ vehicle) travelling at 60, 65 and 50 km·h^−1^, respectively; where the speed limit was 80 km·h^−1^. The road was marked with a broken center line, indicating that it was legal to overtake; however, frequent bends and hills (impairing longitudinal vision), added an element of risk to this maneuver. If participants chose to overtake, the number of overtaking maneuvers performed (OT) and the maximum vehicle speed whilst overtaking (max. OT speed) were recorded. If they did not overtake, the median distance between the participant’s car and the lead vehicle (headway distance) was measured when the participant’s car was first within 50 m of the lead vehicle.

### 2.6. Subjective Ratings

Visual analog scales were used to evaluate subjective mood and arousal [30], intoxication and impairment [25], and perceived driving performance [25]. Each scale was administered via a computerized modifiable software program, Adaptive Visual Analog Scales (AVAS) [31], on a laptop computer. Participants placed the cursor on a 100 mm line with antonyms as anchor points, to indicate how they felt at that time. Questions were adapted from those used previously with demonstrated sensitivity to the impairing effects of alcohol [22,25].

### 2.7. Statistical Analysis

All statistical analyses were performed using SPSS for Windows, Version 23.0 (SPSS Inc., Chicago, IL, USA). All measures were examined for normality and sphericity using the Shapiro–Wilk test and Mauchly’s test, respectively. Paired *t* tests were used to compare baseline conditions (i.e., Stanford Sleepiness Scale, U_SG_ and BGL) across trials. BrAC and BGL measurements obtained pre- and post-Drive 2 were investigated via 2 (treatment) × 2 (time) × 2 (gender) split-plot ANOVAs, where treatment and time were within- and Gender was a between-subject factor(s). Pairwise comparisons (Bonferroni correction factor applied) were performed where significant main effects were present. Paired and independent *t* tests (for within and between-subject analyses, respectively) were used to conduct post hoc comparisons where significant interaction effects were present. An adjusted-alpha level (i.e., *p* = 0.05 divided by the number of tests performed) was used to account for multiple comparisons. Where BrAC was below the detectable level of the breathalyzer instrument (<0.008%) pre-drive (*n* = 3), it was taken as 0.007%; where BrAC was below detectable levels post-drive (*n* = 6), it was taken as the pre-drive BrAC minus the average change in BrAC pre- to post-drive (for a given treatment and gender). If this value was ≥0.008%, BrAC was taken as 0.007% (*n* = 1). Planned comparisons were conducted to test the specific hypothesis that driving tests performed post-alcohol ingestion (i.e., Drive 2) (with or without CHO) would demonstrate impairment in comparison to baseline tests (Drive 1). In addition to the total participant analysis, analysis of driving performance data for participants who had a mean BrAC (pre-post Drive 2) >0.021% on the AS trial was performed. This secondary analysis was conducted to improve measurement sensitivity based on a recent meta-regression, which demonstrated that acute alcohol consumption causes an increase in SDLP during simulated driving, starting from as low as 0.021% BrAC [22]. The statistical analysis for each driving performance variable (i.e., SDLP_1_, SDLP_2_, SDLP_Total_, LC, OT, max. OT speed (for vehicles #1–3) and headway distance) involved split-plot ANOVAs, where Drive and Gender were within- and between-subject factors, respectively. Pairwise and post hoc comparisons were conducted as described previously. Repeated measures intervention effect sizes were calculated as Hedges’ g, where the mean difference between the baseline and the intervention performance score was standardized against the standard deviation of change and corrected for bias due to small sample size [32,33] using a supplementary spreadsheet [34]. Normally distributed subjective ratings were investigated using a 2 (treatment) × 2 (time) ANOVA or paired *t* tests (if no time parameter existed); the Wilcoxon Signed Rank Test was used to investigate non-normally distributed subjective ratings. Statistical significance was accepted at *p* < 0.05. All data are reported as mean ± SD.

## 3. Results

### 3.1. Participant Characteristics

All participants verbally acknowledged compliance to the pre-experimental procedures and provided a breath sample with no detectable alcohol (i.e., BrAC = 0.000%) on arrival at the laboratory. Participants began each trial in a similar state of alertness (as indicated by the Stanford Sleepiness Scale, CHO: 2.1 ± 0.9; AS: 2.1 ± 0.9, *t*(31) < 0.001, *p* = 1.000); euglycemia (CHO: 5.2 ± 0.5 mmol/L; AS: 5.2 ± 0.5 mmol/L, *t*(31) = 1.259, *p* = 0.217); and euhydration (CHO: 1.009 ± 0.007; AS: 1.009 ± 0.006, *t*(31) = 0.212, *p* = 0.834) (Nb. *n* = 6 participants were administered a bolus of plain water due to a pre-trial U_SG_ ≥ 1.024).

### 3.2. Breath Alcohol Responses

**Figure 2:**
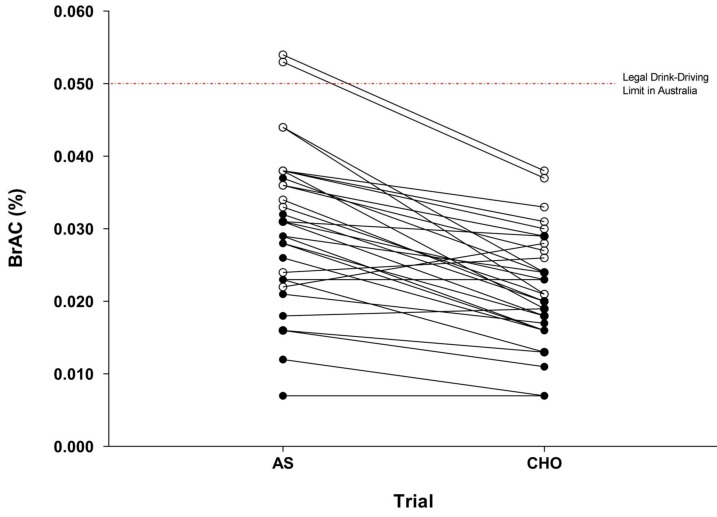
Individual breath alcohol concentrations for AS and CHO trials at the onset of Drive 2 (*n* = 32). AS: artificial sweetener; CHO: carbohydrate; open symbols (ο) are male participants, closed symbols (●) are female participants.

Breath alcohol responses are indicated in Table 1. A two (treatment) × two (time) × two (gender) split-plot analysis of BrAC revealed significant main effects of time (F(1, 30) = 96.4, Pre: 0.026 ± 0.006% vs. Post: 0.020 ± 0.006%); and treatment (F(1, 30) = 66.9, CHO: 0.019 ± 0.006% vs. AS: 0.027 ± 0.006%) (*p* < 0.001). Post hoc comparisons using an adjusted-alpha level (*p* = 0.010) were completed to explore a significant treatment × time interaction (F(1, 30) = 7.60, *p* = 0.010). The results indicate that Drive 2 was performed on the descending limb of the BrAC curve for both the CHO (Pre: 0.022 ± 0.008% vs. Post: 0.017 ± 0.008%) and AS treatments (Pre: 0.030 ± 0.011% vs. Post: 0.023 ± 0.009%), and that the presence of CHO significantly reduced BrAC at both time points (*p* < 0.001). No significant gender × treatment or gender × time interactions were observed (*p* > 0.05). However, a trend for a significant gender × treatment × time interaction was recorded (F(1, 30) = 3.51, *p* = 0.071). Post hoc comparisons using an adjusted-alpha level (*p* = 0.004) indicated similar influences of treatment and time within the male and female subgroups as were noted in the aforementioned comparisons (i.e., decreased BrAC pre vs. post-drive and decreased BrAC with CHO vs. AS) (all *p* < 0.001). The analysis also indicated that females elicited lower BrACs across both treatments and time points (all *p* < 0.001) (Table 1). At an individual level, two male participants provided a breath sample >0.050% BrAC under the AS treatment (0.053; 0.054% BrAC). In contrast, no participant exceeded 0.050% BrAC with the CHO treatment. No females provided a breath sample >0.050% BrAC under either treatment (Figure 2). 

### 3.3. Subjective Ratings

Subjective mood and arousal were evaluated at baseline and the onset of Drive 2 (Shapiro–Wilk *p* > 0.05). Two (treatment) × two (time) analyses revealed a significant main effect of time on participants’ ratings of concentration (F(1, 31) = 28.8); tiredness (F(1, 31) = 18.8); and co-ordination (F(1, 31) = 30.2, *p* < 0.001); such that alcohol consumption decreased feelings of concentration (80 ± 14 mm vs. 69 ± 16 mm), tiredness (71 ± 20 mm vs. 61 ± 17 mm) and co-ordination (82 ± 12 mm vs. 70 ± 16 mm), compared to baseline values. Post hoc comparisons using an adjusted-alpha level (*p* = 0.013) were completed to explore a treatment × time interaction for ratings of excitement (F(1, 30) = 13.7, *p* = 0.001). However, comparisons did not reveal any significant differences across treatments. No other significant main effects or interactive effects were observed. Subjective intoxication and impairment were evaluated at the onset of Drive 2 (Shapiro–Wilk *p* > 0.05). The extent to which participants “felt” the effects of alcohol did not differ significantly due to treatment (*t*(31) = 1.48, *p* = 0.147, (AS: 50 ± 24 mm vs. CHO: 42 ± 25 mm). Although not statistically significant, subjects tended to report lower willingness to drive (*t*(31) = 1.85, *p* = 0.073, AS: 61 ± 27 mm vs. CHO: 68 ± 28 mm); and anticipated increased levels of driving impairment (*t*(31) = 1.99, *p* = 0.056, AS: 38 ± 21 mm vs. CHO: 30 ± 23 mm) under the AS treatment. 

### 3.4. Blood Glucose Responses

A two (treatment) × two (time) × two (gender) split-plot analysis of BGL data indicated a significant main effect of treatment (F(1, 30) = 119, *p* < 0.001), such that BGL was significantly elevated when alcohol was ingested with CHO compared to AS (CHO: 7.3 ± 1.2 vs. AS: 5.1 ± 0.3 mmol·L^−1^). No other significant main effects or interaction effects were observed (all *p* > 0.05). 

### 3.5. Driving Performance

Driving performance data from *n* = 9 participants who provided a mean pre-post Drive 2 BrAC ≤0.021% following the AS treatment were excluded from the analysis of simulated driving performance; the remaining sample comprised *n* = 23 individuals; the mean pre-/post-drive BrACs for this participant subgroup are displayed in Table 2. SDLP_1_, SDLP_2_ and SDLP_Total_ (1 and 2) did not differ significantly due to trial order (*t*(22) = 1.26, *p* = 0.220; *t*(22) = 0.100, *p* = 0.922; *t*(22) = 0.844, *p* = 0.408, respectively), suggesting that learning effects for simulated driving were not evident and did not confound driving performance results between trials.

#### 3.5.1. Vehicle control.

Lateral control data analysis of the total sample (*n* = 32) and the subgroup (*n* = 23) of individuals with BrAC > 0.021% on the AS treatment are provided in Table 3 and Table 4, respectively. A series of two (drive) × two (gender) split-plot analyses failed to detect any significant differences between comparisons (*p* > 0.05). However, a non-significant trend was observed for changes in SDLP_1_ following the AS treatment (*p* ~ 0.085 for both the total and subgroup analyses), suggesting that alcohol tended to impair lateral control in the absence of CHO along the first segment of the roadway. Neither SDLP_2_, SDLP_Total_ nor LC differed significantly between Drives 1 and 2 under either of the treatment conditions (all *p* > 0.05); no significant interactions by gender were observed in these analyses (all *p* > 0.05).

#### 3.5.2. Risky-Driving Behavior 

Data for the risky driving segment of the course (including revised sample sizes based on occurrences for inclusion) are displayed in Table 5. A two (drive) × two (gender) split-plot analysis indicated no significant differences in the number of OT maneuvers performed on Drive 1 vs. Drive 2 under either treatment condition (*p* > 0.05). There was a tendency for male participants to perform more OT maneuvers than females following the CHO treatment during Drive 1 (2.2 ± 1.3 vs. 1.1 ± 1.4, *p* = 0.073) and Drive 2 (2.4 ± 1.3 vs. 1.1 ± 1.5, *p* = 0.042). Male participants also performed more OT maneuvers following the AS treatment than females (Drive 1: 1.9 ± 1.4 vs. 1.6 ± 1.5; Drive 2: 2.4 ± 1.3 vs. 1.3 ± 1.6); however, these differences were non-significant (*p* > 0.05). Analysis of the maximum OT speed was performed when subjects attempted an OT maneuver on both Drive 1 and Drive 2, following a given experimental treatment (i.e., comparisons could not be completed if an OT maneuver was performed on Drive 1, but not Drive 2 within a trial). While the mean maximum OT speed exceeded the signed limit (80 km·h^−1^) on all occasions, two (drive) × two (gender) split-plot analyses failed to identify a significant difference in the maximum OT speed for Drive 1 vs. Drive 2 comparisons in both CHO and AS trials (all *p* > 0.05). Distance headway was not significantly different on Drive 1 vs. Drive 2 following either treatment (*p* > 0.05). Neither maximum OT speed nor headway distance differed significantly due to gender (all *p* > 0.05). 

#### 3.5.3. Perceived Driving Performance

‘Perceived’ driving performance was evaluated on completion of Drive 2 (Nb. Data from *n* = 9 participants who provided a mean pre-post Drive 2 BrAC ≤0.021% following the AS treatment were excluded from these analyses). Subjective ratings were non-normally distributed (*p* < 0.05). Participants rated their overall driving performance (72 mm [11] vs. 74 mm [18]; *p* = 0.820), ability to drive safely (79 mm [15] vs. 74 mm [16]; *p* = 0.475), ability to adhere to speed limits (75 mm [22] vs. 73 mm [30]; *p* = 0.270) and ability to adhere to road rules (81 mm [17] vs. 80 mm [24]; *p* = 0.360) similarly under both the CHO and AS treatments, respectively (median [IQR]).

### 3.6. Participant Blinding and Expectations

Twenty-one participants (66%) correctly identified the trial in which they received the CHO treatment. Seven individuals (22%) were aware that CHO may attenuate peak BrAC when ingested with alcohol, in comparison to an artificial sweetener. Three individuals (9%) were aware that CHO ingestion may enhance cognitive function in fasted individuals. Three participants (9%) reported greater confidence in their driving ability across trials (i.e., from Trial 1 to 2) as a result of familiarization with the test. Six participants (19%) indicated a reason for not overtaking cars along the roadway was because they believed the maneuver was not allowed under Australian road rules. Seven participants (22%) indicated that they deliberately engaged in risky-driving behaviors during the tests; these behaviors included overtaking on a hill with restricted vision (*n* = 6) and speeding around corners (*n* = 1).

## 4. Discussion

This study examined the effects of co-ingesting alcohol (in gender specific doses recommended by NDARC [12] to reduce the acute risk of harm) with CHO or AS on simulated driving performance. Overall, the results obtained partially support our hypothesis, with the presence of CHO in the alcoholic beverage resulting in lower BrAC responses. However, we were unable to detect changes in simulated driving performance following consumption of alcohol; therefore, we did not observe an attenuation of alcohol-induced driving impairment with the CHO based beverage.

Observations from this study are consistent with recent reports indicating a reduction of BrAC in both males and females when alcohol is co-ingested with CHO compared to AS [15,16]. Previous studies have indicated peak BrAC reductions in the magnitude of 18–37%, depending on the quantity of alcohol and CHO provided [14,15,16]. In the present study, the addition of 25 g of CHO to the alcoholic beverage resulted in a mean reduction in BrAC of 30% (males: 31%, females: 29%) at the onset of the driving task. This response was relatively consistent, with 27 of the 32 participants demonstrating reduced BrAC when alcohol was consumed with CHO compared to AS (BrAC was increased for *n* = 3 and identical for *n* = 2). Importantly, while mean BrACs at the onset of driving were below the 0.050% drink-driving limit for both genders in the AS trial (males: 0.036%; females: 0.024% BrAC), two male participants provided a breath sample in excess of 0.050%, thus exceeding the legal drink-driving limit in Australia. Inter-individual variability in alcohol responses has been consistently reported and is often attributed to factors such as differences in body composition, ethnicity and dietary behaviors [13]. Screening (e.g., age, body mass index) and standardization protocols (e.g., for consistency in dietary behavior between trials) were incorporated into this study to limit the influence of these factors. However, the observed variability may be explained by individual factors unable to be controlled (i.e., alcohol drinking history and genetics). This is of importance as these are not considered in population-level alcohol recommendations, questioning the reliability and appropriateness of this advice for broad population use. Current recommendations should highlight the implications of consuming alcohol under different conditions (i.e., when beverages are mixed with CHO or AS); and how this may influence the likelihood of exceeding enforceable drink-driving limits.

Participants in this study indicated no differences in perceived level of intoxication and impairment, irrespective of differences in BrAC between trials [16,17]. Previous studies have reported similar findings where providing larger alcohol doses, elicited higher (>0.070%) BrAC levels [16,35]. Collectively, these results suggest that individuals are unable to detect divergence in physiological responses when a low to moderate acute alcohol dose is mixed with CHO or AS. This has significant implications for individuals who attempt to predict their level of intoxication and impairment as a means of assessing their ability to safely and legally operating a motor vehicle, particularly when consuming alcoholic beverages devoid of CHO. Although not statistically significant, subjects in the present study did tend to report lower willingness to drive and anticipated increased levels of driving impairment after consuming alcohol with AS compared to CHO. This may have been a result of the majority of individuals (66%) correctly identifying the CHO trial and a proportion of these individuals (22%) being aware that CHO can attenuate peak BrAC when ingested with alcohol. These participants tended to be university students currently enrolled in nutrition-related courses. Thus, participants may have made more conservative decisions concerning willingness to drive under the CHO treatment. Whether these results translate into actual behavior in natural environments requires further consideration.

Overall, simulated driving performance was not impaired following consumption of either alcohol treatment in this study. There was a trend (not statistically significant) towards a decrement in lateral vehicle control along the first segment of roadway in the AS, but not in the CHO trial. That is, SDLP increased from baseline during this segment of the driving task by 1.7 cm (*Hedges g* = 0.26). Importantly, this magnitude of change remains below previous reports of clinically relevant increases in SDLP (i.e., ~2.0 cm at 0.050% BrAC) [22]. The lack of effect on driving performance, despite the difference in BrAC, may relate to two methodological factors. Firstly, the timing of the simulated drives resulted in the majority of experimental drives being performed on the descending limb of the biphasic BrAC curve. It has been suggested that alcohol’s deleterious effects on cognitive and motor performance are greater during the ascending rather than descending phase of the curve [36]. Furthermore, cognitive performance may recover to baseline levels during the descending limb [37,38]. Thus, detection of subtle changes in driving performance outcomes in the present study may have been compromised by the timing of performance measurement relative to alcohol administration. Secondly, the study may have been underpowered for assessing a change in performance when the more sensitive BrAC criteria (i.e., BrAC > 0.021%) was applied. Future research should consider replicating this study with changes to the timing of task performance to examine the effects on the ascending limb of the BrAC curve and the inclusion of greater numbers of participants whose alcohol response is more likely to produce perturbations in simulated driving performance.

It is important to recognize that simulated driving research may be limited in its generalizability and real-world application [39]. Thus, results from the present study may not directly translate to on-road driving behavior. Task complexity is also a key determinant of detecting alcohol-induced impairment in simulated driving research [22], with higher levels of impairment reported as the complexity of the task increases [11,38]. Complex tasks involving perception and visual processing are particularly susceptible to alcohol-induced impairment [40]. With respect to driving simulation, task complexity can be increased by adding environmental stimuli, complex roadway geometry and secondary tasks [38]. The present study employed simple roadway geometry (i.e., road curvature with large radii), which has been shown to require lower levels of driver skill and be less susceptible to alcohol-induced impairment [41]. The driving scenario also lacked secondary tasks that require drivers to divide attention. Thus, detecting subtle changes in driving performance in response to the different alcohol treatments in the present study may have been limited by the characteristics of the employed driving task and lack of sensitivity in the performance measure.

Despite substantial differences in BrACs in the present study, no significant gender differences were observed for any of the driving performance parameters. Furthermore, ratings of perceived driving performance were comparable between genders and were generally high, irrespective of treatment. These results suggest that provision of alcohol in doses corresponding to NDARC recommendations for reduced risk of harm may be appropriate with respect to limiting the impact on driving performance. Whether the same results are observed when equivalent doses of alcohol (with and without CHO) are provided to males and females requires consideration, particularly as some findings suggest that females demonstrate greater levels of driving impairment than males at equivalent BrACs [42]. It is important to note, however, that gender comparisons in this study may be underpowered as participant numbers were not determined on the basis of conducting gender comparisons. Thus, a larger sample size is likely to be required to determine significant group differences. 

## 5. Conclusions

In summary, results of this investigation support previous research demonstrating that BrACs are attenuated when alcohol is co-ingested with CHO as opposed to AS, in both males and females. Furthermore, ingesting alcohol in quantities advised by the NDARC to reduce the risk of alcohol-related harm resulted in no detectable impairment in simulated driving performance. Thus, we were unable to observe an attenuation of alcohol-induced driving impairment when alcohol was co-ingested with CHO compared to AS. Despite this, our results highlight the large variability in individual BrAC responses when alcohol is consumed and suggest that the likelihood of exceeding the legal driving BrAC is increased when alcohol is consumed without additional CHO.

## Figures and Tables

**Table 1:** Mean breath alcohol responses by treatment, time and gender (*n* = 32).

	BrAC Pre-Drive 2 (%)		BrAC Post-Drive 2 (%)	
	AS	CHO	AS	CHO
Males	0.036 ± 0.010	0.025 ± 0.007	0.028 ± 0.008	0.022 ± 0.006
Females	0.024 ± 0.009	0.017 ± 0.008	0.018 ± 0.006	0.010 ± 0.008

AS: artificial sweetener; BrAC: breath alcohol concentration; CHO: carbohydrate. Values are Mean ± SD.

**Table 2:** Mean pre-/post-drive BrACs by treatment and gender (based on subgroup of *n* = 23 participants where mean pre-/post-drive BrAC on the AS treatment was >0.021%).

	Mean Pre-/Post-Drive BrAC (%)	
	AS	CHO
Males (*n* = 14)	0.034 ± 0.007	0.025 ± 0.006
Females (*n* = 9)	0.026 ± 0.003	0.018 ± 0.004
Overall (*n* = 23)	0.031 ± 0.007	0.022 ± 0.006

AS: artificial sweetener; BrAC: breath alcohol concentration; CHO; carbohydrate. Values are Mean ± SD.

**Table 3:** Lateral control driving performance data (*n* = 32).

	AS Treatment				CHO Treatment			
	Drive 1	Drive 2	*p*	Hedges’ *g*	Drive 1	Drive 2	*p*	Hedges’ *g*
SDLP_1_ (cm)	24.7 ± 5.2	26.0 ± 6.3	0.087	0.22	26.1 ± 7.9	26.0 ± 6.8	0.899	0.01
SDLP_2_ (cm)	36.1 ± 9.4	34.2 ± 8.1	0.144	0.21	32.7 ± 7.4	33.5 ± 7.7	0.529	0.10
SDLP_Total_ (cm)	32.1 ± 7.2	31.4 ± 6.7	0.472	0.09	30.7 ± 6.6	31.1 ± 6.7	0.672	0.07
LC (*n*)	12.3 ± 7.4	11.8 ± 6.8	0.590	0.07	11.5 ± 7.6	13.3 ± 10.1	0.085	0.18

AS: artificial sweetener; CHO; carbohydrate; LC: lane crossings; SDLP: standard deviation of lane position. Values are Mean ± SD.

**Table 4:** Lateral control driving performance data (based on subgroup of *n* = 23 participants where mean pre-/post-drive BrAC on the AS treatment was >0.021%).

	AS Treatment				CHO Treatment			
	Drive 1	Drive 2	*p*	Hedges’ *g*	Drive 1	Drive 2	*p*	Hedges’ *g*
SDLP_1_ (cm)	23.8 ± 4.3	25.5 ± 6.7	0.085	0.26	24.6 ± 0.62	24.9 ± 5.8	0.776	0.05
SDLP_2_ (cm)	35.4 ± 8.3	33.4 ± 5.6	0.275	0.27	32.2 ± 0.60	33.0 ± 5.7	0.469	0.13
SDLP_Total_ (cm)	31.1 ± 8.2	31.1 ± 6.7	0.593	0.00	30.3 ± 0.58	30.6 ± 4.8	0.688	0.05
LC (*n*)	12 ± 8	11 ± 8	0.376	0.12	11 ± 8	12 ± 10	0.773	0.10

AS: artificial sweetener; CHO; carbohydrate; LC: lane crossings; SDLP: standard deviation of lane position. Values are Mean ± SD.

**Table 5:** Risky driving behavior (based on subgroup of *n* = 23 participants where mean pre-post Drive 2 BrAC on the AS treatment was >0.021%).

	AS Treatment					CHO Treatment				
	*n*	Drive 1	Drive 2	*p*	Hedges’ *g*	*n*	Drive 1	Drive 2	*p*	Hedges’ *g*
OT (*n*)	-	1.7 ± 1.5	1.8 ± 1.4	0.479	0.07	-	1.7 ± 1.4	1.7 ± 1.4	0.528	0.00
Max. OT Speed #1 (km·h^−1^)	14	86.0 ± 6.1	87.7 ± 6.6	0.366	0.26	14	84.9 ± 10.1	86.9 ± 7.1	0.512	0.22
Max. OT Speed #2 (km·h^−1^)	12	91.1 ± 7.1	90.0 ± 5.8	0.460	0.16	13	90.3 ± 12.4	92.2 ± 6.9	0.485	0.16
Max. OT Speed #3 (km·h^−1^)	12	82.8 ± 5.8	83.6 ± 7.7	0.558	0.11	13	84.1 ± 12.1	83.1 ± 8.9	0.492	0.08
Headway distance (m)	8	38.9 ± 18.7	37.2 ± 9.4	0.831	0.11	9	45.6 ± 26.2	33.9 ± 15.8	0.055	0.39

AS: artificial sweetener; CHO; carbohydrate. OT: overtaking maneuver. Values are Mean ± SD.
